# Tibial tunnel enlargement is affected by the tunnel diameter-screw ratio in tibial hybrid fixation for hamstring ACL reconstruction

**DOI:** 10.1007/s00402-022-04408-2

**Published:** 2022-03-14

**Authors:** Andreas Flury, Linda Wild, Manuel Waltenspül, Christoph Zindel, Lazaros Vlachopoulos, Florian B. Imhoff, Sandro F. Fucentese

**Affiliations:** Orthopaedic Department, Balgrist University Hospital, University of Zurich, Forchstrasse 340, 8008 Zurich, Switzerland

**Keywords:** Tunnel diameter, Anterior cruciate ligament reconstruction, Interference screw, Hybrid fixation

## Abstract

**Introduction:**

There is no evidence on screw diameter with regards to tunnel size in anterior cruciate ligament reconstruction (ACLR) using hybrid fixation devices. The hypothesis was that an undersized tunnel coverage by the tibial screw leads to subsequent tunnel enlargement in ACLR in hybrid fixation technique.

**Methods:**

In a retrospective case series, radiographs and clinical scores of 103 patients who underwent primary hamstring tendon ACLR with a hybrid fixation technique at the tibial site (interference screw and suspensory fixation) were obtained. Tunnel diameters in the frontal and sagittal planes were measured on radiographs 6 weeks and 12 months postoperatively. Tunnel enlargement of more than 10% between the two periods was defined as tunnel widening. Tunnel coverage ratio was calculated as the tunnel diameter covered by the screw in percentage.

**Results:**

Overall, tunnel widening 12 months postoperatively was 23.1 ± 17.1% and 24.2 ± 18.2% in the frontal and sagittal plane, respectively. Linear regression analysis revealed the tunnel coverage ratio to be a negative predicting risk factor for tunnel widening (*p* = 0.001). The ROC curve analysis provided an ideal cut-off for tunnel enlargement of > 10% at a tunnel coverage ratio of 70% (sensitivity 60%, specificity 81%, AUC 75%, *p* < 0.001). Patients (*n* = 53/103) with a tunnel coverage ratio of < 70% showed significantly higher tibial tunnel enlargement of 15% in the frontal and sagittal planes. The binary logistic regression showed a significant OR of 6.9 (*p* = 0.02) for tunnel widening > 10% in the frontal plane if the tunnel coverage ratio was < 70% (sagittal plane: OR 14.7, *p* = 0.001). Clinical scores did not correlate to tunnel widening.

**Conclusion:**

Tibial tunnel widening was affected by the tunnel diameter coverage ratio. To minimize the likelihood of disadvantageous tunnel expansion—which is of importance in case of revision surgery—an interference screw should not undercut the tunnel diameter by more than 1 mm.

## Introduction

Tibial and femoral bone tunnel widening following ACL reconstruction is a well-known phenomenon. Although most studies have not reported any negative associations to laxity or other clinical outcome scores [1–4], excessive tunnel expansion might be a severe disadvantage for revision surgery because of a required two-stage reconstruction approach [5, 6]. Unfortunately, failure and recurrent instability rate of ACL reconstruction is reported between 10% and 15% leading to a large number of revision ACL reconstruction, mainly affecting young athletes [7, 8]. Therefore, it is important to understand possible causes to avoid tunnel enlargement.

Although the exact pathophysiology of tunnel widening remains unclear, it has been postulated to be the result of a combination of both biological and mechanical factors [4]. Described biological factors are synovial fluid propagation into the tunnels, inferior bone quality and non-specific inflammatory response caused by localized bone necrosis due to thermogenic effects with the drilling process [9]. The type of graft fixation and graft motion within the tunnels were described as mechanical factors. In contrast to bone-tendon-bone transplants, the artificial tendon–bone interface of hamstring grafts allows micromovements [10] and results in higher rates of tunnel enlargement [3, 11]. Furthermore, graft fixation with interference screws was reported to be associated with enlarged bone tunnels when compared to extracortical fixation techniques [11–14].

Hybrid fixation technique combines both aperture and extracortical fixation, and was shown to increase fixation strength and stiffness in biomechanical and clinical studies [15–18]. The graft is mainly fixated with the suspensory device, and, therefore, allows leeway with regard to the screw diameter. In fact, there is no standard of how the interference screw thickness should be chosen in relation to the tunnel diameter. To press the graft against the wall and, therefore, promote healing, usually a screw a few millimeters smaller compared to the tunnel diameter is used [19]. Moreover, the distance between the graft fixation points is reduced with an interference screw, resulting in decreased longitudinal graft motion (bungee cord effect) [12, 20, 21]. However, a considerable mismatch between the screw to the tunnel diameter might produce a mechanically unfavorable environment in that increased sagittal movements of the graft occur during flexion/extension of the knee (windshield wiper effect). Thus, tunnel enlargement might be the result of the stress exerted on the wall in the direction where the graft runs and pulls [22].

There is no evidence if a small or large screw diameter compared to tunnel diameter is favorable regarding tunnel widening, or whether this is negligible because all bone tunnels become slightly larger within the first 6 months after ACL reconstruction. Therefore, it was the study's hypothesis that the smaller the proportion of the drill channel covered by the screw, the greater the subsequent tunnel expansion.

## Methods

### Inclusion and exclusion criteria

**Fig. 1:**
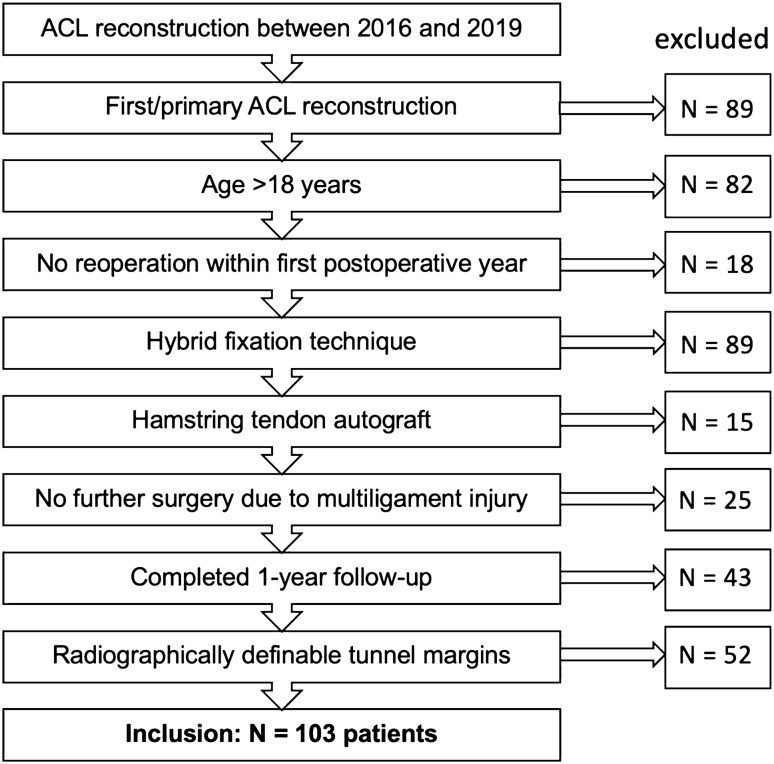
Flowchart of patients' recruitment

For this retrospective study, all patients who underwent primary arthroscopic ACL reconstruction using hamstring autograft tendon between 2016 and 2019 with a minimum follow-up of one year were included. Patients with additional injuries to knee ligaments were excluded when further surgery on the ipsilateral knee was needed (*n* = 25). Further exclusion criteria were: age < 18 years (*n* = 82), infection, reoperation within one year after primary ACL reconstruction due to a complication of any kind (*n* = 18), ACL re-rupture (*n* = 44), secondary ACL reconstruction (*n* = 89), and a missed 1-year follow-up (*n* = 43). To account for confounding factors, only hybrid fixated hamstring autograft reconstructions were included in the analysis (Fig. 1). Concomitant meniscal tears and/or chondral disruptions requiring surgical repair were no exclusion criteria. The radiolucent area of the tibial tunnel is not always visible on immediate postoperative radiographs [23]. Accordingly, due to the indefinability of the tibial tunnel margins, further 52 cases had to be excluded.

Surgery was performed by > 6 experienced knee surgeons, and each surgeon freely chose the size of the screw at his own discretion. Therefore, a heterogeneous composition of the study cohort can be assumed.

12 months after surgery IKDC and Tegner score were recorded.

### Surgical technique

The ACL was reconstructed using a hamstring autograft. The semitendinosus and (if needed) gracilis tendons were harvested through a small incision over the Pes Anserinus. All femoral tunnels were drilled through an anteromedial portal with focus put on establishing anatomic ACL graft position and orientation. The tibial tunnel was drilled in 45° of flexion with s specific guide. The end thickness of the tibial tunnel was achieved either with the drill or with a dilator, and always corresponded to the tibial-sided graft thickness. The grafts were inserted via the tibial tunnel. At the femoral side, the grafts were secured with a button (Fliptack, Karl Storz). At the tibial side, a hybrid fixation was used: In addition to the extracortical fixation of the suspensory device (Endotack, Karl Storz), a 23 mm long bioresorbable interference screw (Mega fix, Karl Storz) with the knee positioned in 20° of flexion [24] was inserted, approximately 20–30 mm within the tunnel (in a way that it was not visible arthroscopically). Previously, the graft was tensioned with maximal manual power [19].

### Tunnel coverage ratio

To describe the percentage of the drill channel that is covered by the screw, the tunnel coverage ratio was introduced. The tunnel coverage ratio was defined as the radius square of the interference screw divided to the radius square of the tibial tunnel (Table 1). In this study, the MegaFix interference screw by Storz (Tuttlingen, Germany) was investigated, for which the diameter corresponds to the outer diameter of the screw.

**Table 1:**
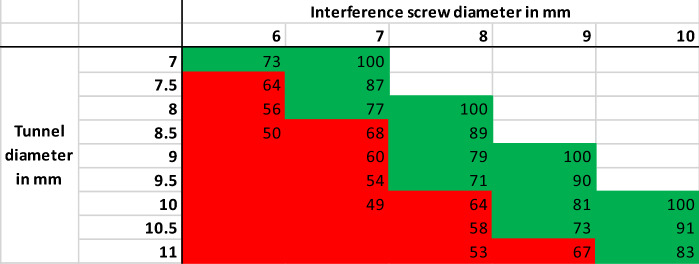
Tunnel coverage ratio

### Rehabilitation protocol

The knee was first immobilized in an extended position for 5–7 days with immediate postoperative active and passive range of motion exercises. For a total of 6 weeks, all patients used a hinged knee brace with partial weight-bearing starting from the first week, with full weight-bearing at 6 weeks. Jogging and running were allowed three months postoperatively. Return to athletic sports with pivoting elements was allowed not earlier than 9 months after ACL reconstruction.

For concomitant chondral and meniscal repair, adjustments of the rehabilitation protocol in terms of range of motion restriction were made: 60° of flexion was allowed the first two weeks, followed by 90° for weeks 3 and 4, and 120° for weeks 5 and 6.

### Radiographic evaluation

Tunnel enlargement primarily occurs within the tibial tunnel, and only minimally within the femoral tunnel, as reported in the literature [1, 25, 26]. Therefore, only the tibial tunnel was investigated in the current study.

**Fig. 2:**
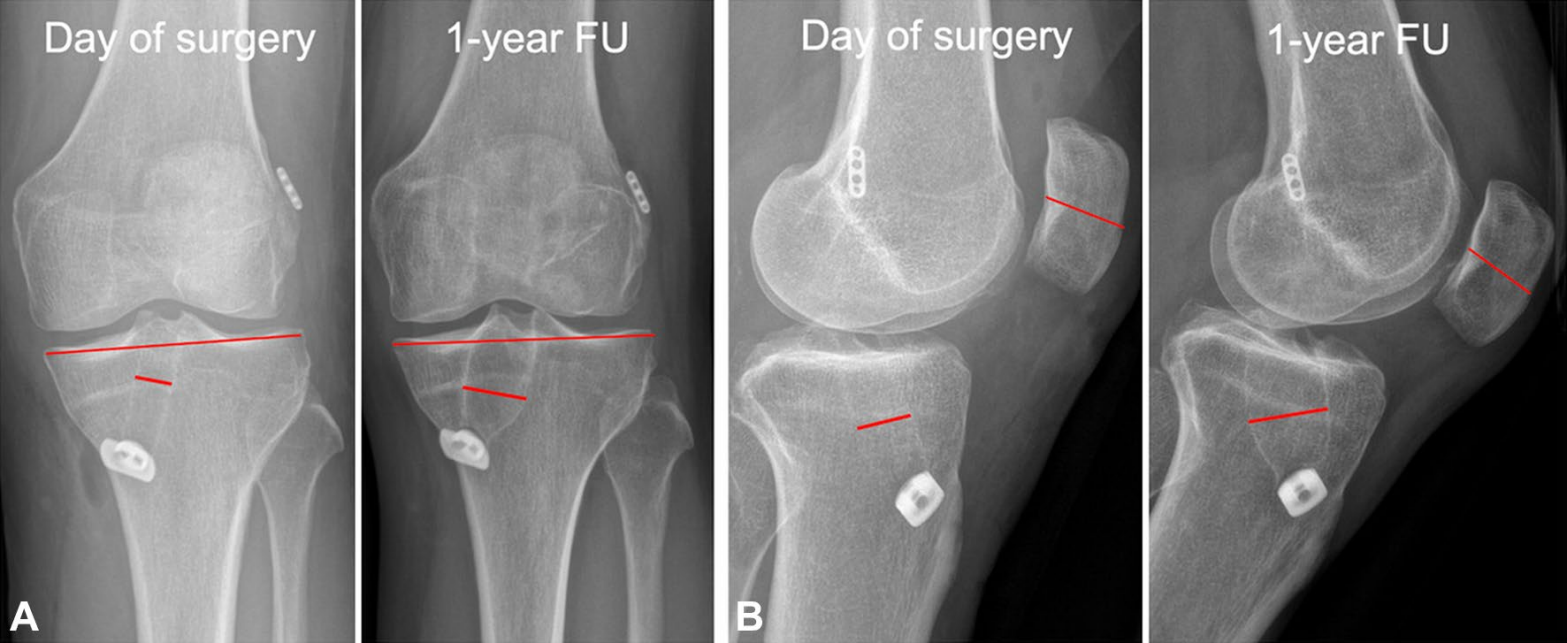
The sclerotic margins of the tibial bone tunnel were measured at the widest dimension of the tunnel in the frontal and sagittal plane, and compared to the first postoperative radiograph after correction for radiographic magnification. A percentage change between the two periods was defined as percentage tunnel enlargement in diameter, and a change of more than 10% was defined as an enlarged tunnel

Each subject had an antero-posterior (in 15° of weight-bearing flexion) and a lateral digital radiograph of the knee immediately postoperatively and at 12 months. Because of a reported considerable interrater error associated with bone tunnel measurement [23], two examiners measured all patients and the average of both readers was calculated. Tunnel measurements were taken at the widest point of the tibial tunnel in each plane, perpendicular to the long axis of the tunnel [23]. According to previous studies [2, 3, 21, 26, 27], each diameter of bone tunnel was calculated as a percentage to the maximum joint width of the proximal tibia in the antero-posterior (AP) view, or a percentage to the maximum diameter of the patella in the lateral view (Fig. 2). A percentage change between the two periods was defined as percentage tunnel enlargement in diameter. Moreover, absolute values were noted after correction for radiographic magnification [26]. To determine the incidence of tunnel enlargement, a percentage diameter change of more than 10% was defined as an enlarged tunnel [2, 3, 21].

### Statistical analysis

A previous study reported a percentage tunnel widening in hamstring ACL reconstruction of 24.3 ± 15.9% after 1 year [3]. According to a pilot study, the tunnel widening of patients with a high tunnel coverage ratio was approximately 10% less than in patients with a greater tunnel/interference screw diameter mismatch. Based on these data (anticipated means 20 ± 16 and 30%), a minimum required sample size of 80 for a desired statistical power of 0.8 was calculated.

Descriptive analyses of patient characteristics were performed with use of means and standard deviations for continuous variables and with frequencies and percentages for discrete or dichotomous variables. All parameters were tested with the Kolmogorov–Smirnov test for normality. Statistical comparisons were performed using the chi-squared test and unpaired Student *t* test. A linear regression analysis was performed to represent the linear correlation of the tunnel coverage ratio and tibial tunnel widening. In a multiple regression analysis, other risk factors such as BMI, sex, age, rehabilitation protocol (full versus partial weight-bearing), and tunnel drilling versus tunnel dilatation were investigated. The Receiver-Operator Characteristic (ROC) curve was used to identify the best cut-off value of the tunnel coverage ratio, which would have predicted an optimal or a non-optimal outcome. For the ROC analysis optimal outcome was considered a tunnel enlargement of < 10%. Otherwise, the outcome was considered as non-optimal. The patients were then divided into two groups according to the cut-off value in ROC curve (group 1: tunnel coverage ratio > 70%, group 2: tunnel coverage ratio < 70%), and relative and absolute tibial tunnel enlargement, as well as demographics factors were then compared between groups. A binary logistic regression model was used to calculate odds ratios (OR). All statistical tests were two-sided, and *p* value of < 0.05 was considered statistically significant. All analysis was performed with SPSS (version 23.0; IBM SPSS Statistics).

## Results

Finally, of *n* = 516, a total of 103 patients (male: 46, female: 57) with an average age of 32 years (range, 18–52) met the inclusion criteria for the current study. Mean BMI was 24.2 ± 3.5 kg/m^2^. The mean IKDC and Tegner scores 1 year after surgery were 87 ± 12 and 5.5 ± 2, respectively.

### Radiographic outcomes

Average tunnel widening 12 months after surgery was 23.1 ± 17.1% (2.6 ± 2 mm) and 24.2 ± 18.2% (2.7 ± 2 mm) in the frontal and sagittal plane, respectively. The incidence of tibial tunnel enlargement of > 10% in the frontal and sagittal plane were both 80%. Tunnel coverage ratio was found to be a negative predicting risk factor for tunnel widening (negative coefficient 0.314, *p* = 0.001). In a multiple regression analysis, no correlation of tunnel widening was found to other risk factors such as BMI, sex, age, rehabilitation protocol, and if the final thickness of the tibial tunnel was achieved by drilling or stepwise dilatation (all n.s.).

### ROC analysis

**Fig. 3:**
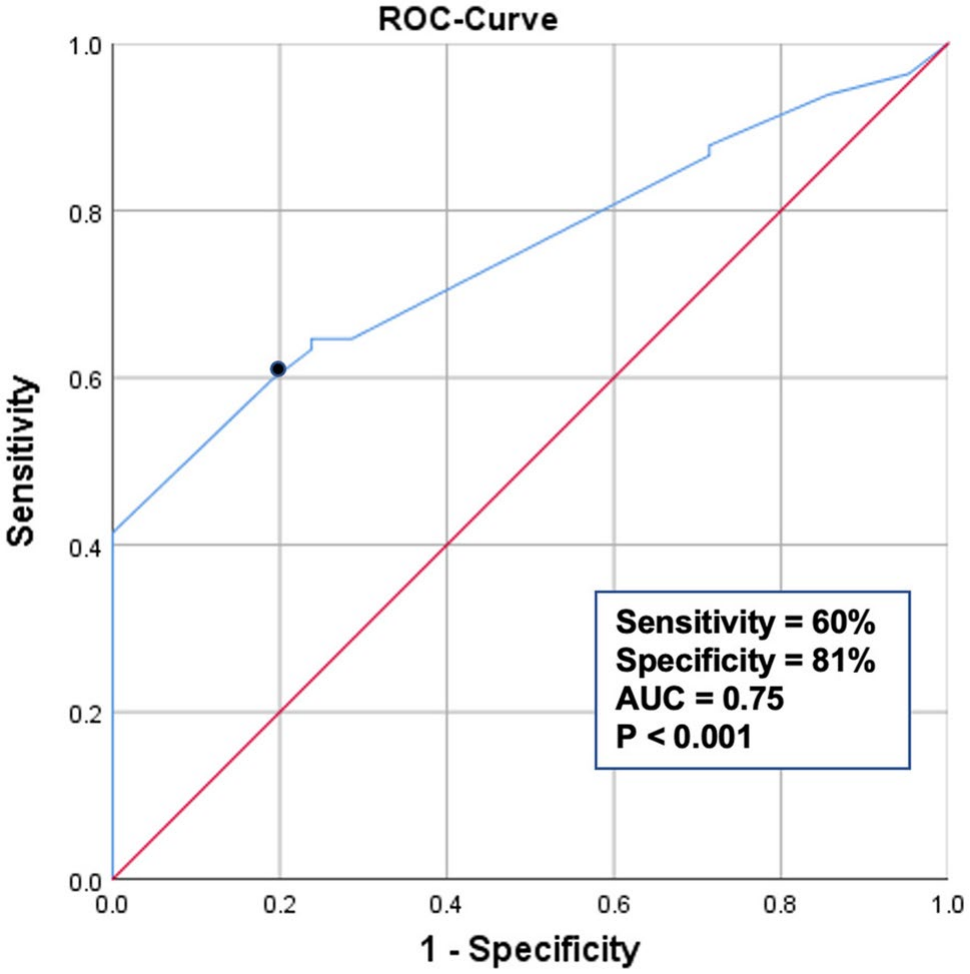
The results of the ROC analysis. With a sensitivity of 60%, a specificity of 81%, and an area under the curve of 75% (*p* < 0.001), a tunnel coverage ratio of 70% was the ideal cut-off for tunnel enlargement of > 10% in the frontal plane one year after surgery (black dot)

The ROC curve analysis provided an ideal cut-off for tunnel enlargement of > 10% in the frontal plane at a tunnel coverage ratio of 70%, with a sensitivity of 60%, a specificity of 81%, and an area under the curve of 75% (*p* < 0.001) (Fig. 3). Identically to the frontal plane, best characteristics for tunnel enlargement of > 10% in the sagittal plane were found at a tunnel coverage ratio of 70% (sensitivity of 63%, specificity 87%).

### Analysis of groups according to tunnel coverage ratio cut-off value

Almost half of patients (*n* = 50) had a tunnel coverage ratio of > 70% (mean 0.8 ± 0.1), whereas 53 patients had a ratio of < 70% (mean 0.6 ± 0.1). No significant difference was observed regarding demographics (Table 2). Relative and absolute tibial tunnel enlargement in both planes were significantly higher in the group with a tunnel coverage ratio < 70% (both *p* < 0.0001): The difference was 15% or 1.5 mm. The binary logistic regression showed significant OR for tunnel widening > 10% if the tunnel coverage ratio was < 70% (frontal plane: OR 6.9, *p* = 0.002; sagittal plane: OR 14.7, *p* = 0.001). Interestingly, IKDC after 1 year was 5 points better in group 2 with tunnel enlargement compared to the group without tunnel enlargement (*p* = 0.04). The difference of the preoperative to postoperative Tegner scores was however better in group 1 (n.s.).

**Table 2:** Analysis of groups generated according to the tunnel coverage ratio cut-off value of 70%

	Tunnel coverage ratio		*p* value
	> 70%	< 70%	
Demographical parameters			
* N*	50	53	
Age (years)	31.2 (7.8)	31.7 (9.1)	0.8
BMI (kg/m^2^)	24 (3.7)	24.4 (3.3)	0.6
Gender			
Male	21 (42%)	25 (47%)	0.6
Female	29 (58%)	28 (53%)	0.6
Radiographical parameters			
Tunnel coverage ratio	0.8 (0.1)	0.6 (0.1)	** < 0.0001**
Incidence of tunnel widening			
AP view	33 (66%)	49 (93%)	** < 0.001**
Lateral view	30 (60%)	50 (94%)	** < 0.0001**
Tunnel widening (%) after 1 year			
AP view	15.4 (13.1)	30.4 (17.5)	** < 0.0001**
Lateral view	17.4 (12.8)	30.5 (20.4)	** < 0.0001**
Tunnel widening (mm) after 1 year			
AP view	1.8 (1.5)	3.4 (2)	** < 0.0001**
Lateral view	2.0 (1.4)	3.5 (2.2)	** < 0.0001**
Clinical parameters			
* N*	41	50	
IKDC after 1 year	84.4 (12.5)	89.6 (11.3)	**0.043**
Difference preop–postop Tegner score	5.6 (1.5)	6.5 (1.7)	0.8

## Discussion

The most important finding of this study was that tibial tunnel widening after primary ACL reconstruction with hybrid fixation is affected by the tunnel diameter covered by the interference screw: A tunnel coverage ratio of > 70% was beneficial regarding tibial tunnel widening.

Previous studies reported enlarged bone tunnels with the insertion of interference screws when compared to other fixation techniques [11–14]. To be specific, Buelow et al. [12] investigated a purely extracortical fixation technique (with an Endobutton femorally, and two no. 6 Ethibond sutures over a washer tibially) and found a significantly increased tibial tunnel area during the first 6 months. However, it then decreased from 65% tunnel expansion to 47%, while graft fixation with interference screw stabilized at a higher 75%. Hybrid fixation technique combines both aperture and extracortical fixation, and was shown to increase fixation strength and stiffness in biomechanical and clinical studies [15–18]. The graft is mainly fixed with the suspensory device. Therefore, in terms of stability, it is not necessary that the interference screw matches the tunnel diameter. However, the screw presses the graft against the tunnel walls for better healing. Nevertheless, there is no consensus on how thick this screw should be. In case of a too small screw, the graft might be eccentrically positioned and not everywhere in contact with the osseous tunnel walls. This could represent an unfavorable mechanical environment that permits sagittal graft motion (so-called windshield wiper effect). Therefore, it was our hypothesis that an undersized tunnel coverage by the tibial screw leads to subsequent tunnel enlargement, which is of clinical importance in case of revision.

And indeed, the newly introduced tunnel coverage ratio accurately predicted an enlarged tunnel > 10% after ACL reconstruction (sensitivity 60%, specificity 81%, *p* < 0.001). If the tunnel coverage ratio was < 70%, OR for tunnel enlargement > 10% in the frontal and sagittal plane was 6.9 and 14.7, respectively. Moreover, if a tunnel enlargement of > 20% is suggested to be more clinically relevant than > 10%, then tunnel coverage ratio of 70% remained the ideal cut-off (sensitivity 70%, specificity 73%, AUC 73%, *p* < 0.001).

This is of clinical relevance in that the surgeon should not be tempted to choose—for fear of graft laceration—a screw that is too small. Against instinct, the tunnel coverage ratio should be 70% or even more. Tibial tunnel width is achieved either with the drill bit (in 1 mm steps) and/or with the dilator (in 0.5 mm steps). Therefore, to not undercut the tunnel diameter by more than 1 mm, a screw diameter of 8 mm but not 7 mm should be chosen in case of a tunnel diameter of 8.5 mm. Because regardless of whether the surgeon is an advocate of a single- or two-stage procedure, revision surgery is way easier if the old tunnels can be reused and no bone grafting is necessary [28].

Other risk factors for tunnel enlargement were reported. Tajima et al. found that a too aggressive rehabilitation protocol resulted in early stress to the graft-bone interface [21, 27]. The rehabilitation protocol was—whether a concomitant meniscus was repaired or not—adapted in our study, but still very similar. Therefore, no conclusive statement can be made in this regard. Overall, except for the tunnel coverage ratio, no other risk factors for tunnel enlargement could be found in the current study. Despite potential increased grip of screws due to trabecular bone compression after tunnel widening with a dilator, no influence on tunnel widening was found.

As reported previously, tunnel widening does not affect short-term clinical outcomes after ACL reconstruction [5, 11]. Unexpectedly, IKDC was significantly better in the group with more pronounced tunnel expansions in the current study. This finding is not explainable. Nevertheless, these clinical scores do not specifically account for graft laxity. Moreover, it is possible that tunnel widening might not be associated with worse clinical 1-year scores, but with recurrence of abnormal laxity in long-term. Therefore, tunnel enlargement might still become clinically relevant due to graft failure [22]. Re-examination of these scores at later time points might reveal different findings. However, the consequence of tunnel widening after ACL reconstruction is foremost of importance in case of revision surgery.

This study has limitations. First, the tibial tunnel is not always visible on radiographs and approximately 30% of the study population had to be excluded. However, this problem has already been reported to the same extent [23], and our required sample size was still met. For this reason, computed tomography (CT) scan would be more appropriate for detection and measurement of bone tunnel width, as well as for tunnel angles and the course of the graft. Therefore, future research with CT data is needed regarding this matter. Moreover, continuous CT follow-up examinations could also give further answers regarding graft healing and the exact timing of tunnel enlargement. Because so far it is still unclear if, in comparison to bone-patellar tendon-bone grafts, soft tissue grafts (hamstring grafts) reliably heal. Next, there is a considerable interrater error associated with bone tunnel measurement [23]. To address this problem, radiographic assessment was done by two senior orthopaedic residents and the average of both readers was taken. If the values of both observers were too different, the case was discussed among the co-authors to reach consensus. Moreover, with 23.1% versus 20.9% in the frontal, and 24.2% versus 25.5% in the sagittal plane, tibial tunnel expansion in our cohort was similar compared to other study cohorts [26]. At last, no statement regarding interference screw fixation of a bone plug ACL graft is possible. Future studies need to explore if there is a difference regarding tunnel widening in case of patellar bone plug fixation in press-fit technique or with an interference screw (gold-standard) [29], or in case of foreign material free ACL reconstruction (with a cortical-cancellous bone cylinder sutured into the hamstring autograft) [30].

## Conclusion

Tibial tunnel widening was affected by tunnel coverage. To minimize the likelihood of disadvantageous tunnel expansion—which is foremost of importance in case of revision surgery—an interference screw should not undercut the tunnel diameter by more than 1 mm.
